# The effects of 8 weeks of functional strength training and blood flow restriction training on lower limb muscle strength, maximal power, and movement quality in male sprinter college athletes

**DOI:** 10.3389/fphys.2026.1798606

**Published:** 2026-05-12

**Authors:** Ji Zhu, Jiale Wang, Huangkun Chen, Ming Li, Yanlin Wang, Yihan Hu

**Affiliations:** 1 School of Physical Education and Sports Science, Fujian Normal University, Fuzhou, China; 2 School of Physical Education, Fuyang Normal University, Fuyang, China; 3 School of Engineering, Xinxiang Institute of Engineering, Xinxiang, China; 4 Institute of Metabolic Science (IMS) Epidemiology Unit, University of Cambridge School of Clinical Medicine, Cambridge, United Kingdom

**Keywords:** blood flow restriction, explosive power, functional strength training, lower limb muscle strength, movement quality, sprinters

## Abstract

**Objective:**

Functional strength training (FST) combined with blood flow restriction (BFR) offers additional benefits in older adults and injured patients, but evidence in athletes is limited. This study aimed to determine whether adding BFR to FST improves lower limb muscle strength, power, and movement quality in male college sprinters.

**Methods:**

Twenty−eight male college sprinters were randomly assigned to an FST−BFR group (n = 14, age 20.17 ± 0.65 years) or an FST group (n = 14, age 19.98 ± 0.39 years). Both groups performed the same FST program for 8 weeks (3 sessions/week). The FST−BFR group wore cuffs set at 50% of arterial occlusion pressure. Pre− and post−intervention assessments included isokinetic knee strength at 60°·s^-^¹ and 300°·s^-^¹, countermovement jump (CMJ), squat jump (SJ), Wingate 30−s anaerobic test [average power (AP), peak power (PP), minimum power (MinP)], functional movement screen (FMS), and lower quarter Y−balance test (YBT). Data were analysed using two−way repeated−measures ANOVA (parametric) and Wilcoxon/Mann−Whitney tests (non−parametric).

**Results:**

Both groups improved over time in most measures (p < 0.05). A significant group × time interaction was observed for AP (F = 80.51, p < 0.001, η²_p_ = 0.756), with the FST−BFR group showing a greater improvement (mean change +92.99 W vs. +34.72 W). For PP, 300°·s^-^¹ knee flexor strength, CMJ, and SJ, significant group × time interactions were found (p < 0.05), but simple effects analysis did not detect significant between−group differences at post−intervention (p > 0.05). No significant interactions were found for YBT, FMS, or other isokinetic variables (all p > 0.05).

**Conclusion:**

Eight weeks of FST, with or without BFR, was associated with significant improvements over time in isokinetic strength, movement quality, and dynamic balance in male college sprinters; however, in the absence of a no-intervention control group, these improvements cannot be directly attributed to the FST programme itself. The addition of BFR provided an additional benefit only for average power during the Wingate test.

## 1 Introduction

Lower limb muscle strength, explosive power, and movement quality are key determinants of a sprinter’s competitive performance (Seitz et al., 2014; Shuai et al., 2025). While traditional high-intensity resistance training effectively improves muscle strength and hypertrophy, its high mechanical load may increase injury risk and complicate integration with sport-specific training during the competitive season (Kraemer et al., 2002; Balagué et al., 2024). Therefore, exploring alternative training methods that promote muscular adaptations while reducing injury risk is of great practical importance for sprinters.

Functional Strength Training (FST) as an emerging training paradigm, emphasizes compound movements involving multiple joints, multiple planes, and unstable support surfaces. It aims to improve strength while simultaneously enhancing coordination, balance, and motor control (Guler et al., 2021). A recent systematic review confirmed that functional training significantly improves physical and technical performance in athlete populations (Xiao et al., 2025). A controlled trial in adolescent sprinters reported that 8 weeks of functional training produced substantial gains in agility and coordination, whereas traditional training primarily contributed to strength gains and sprint time (Liu et al., 2025). Similarly, a systematic review on basketball players found that functional training significantly improves muscle strength, linear speed, balance, and muscular endurance (Cao et al., 2024).However, because FST typically uses low external loads to preserve movement complexity and quality, its metabolic load and hypertrophic stimulus may be limited (Brogno, 2025; Ozaki et al., 2016).

Blood Flow Restriction (BFR) training simulates the metabolic environment of high-load training by applying appropriate pressure to the proximal limbs to partially block venous return while maintaining arterial blood flow, thus mimicking the high-load training environment during low-load training (Scott et al., 2014). The main physiological mechanisms of BFR training include tissue hypoxia, accumulation of metabolic products, cell swelling, and preferential recruitment of fast-twitch muscle fibres (Hwang and Willoughby, 2019; Pearson and Hussain, 2015). A scoping review concluded that BFR training improves muscle strength and hypertrophy across all age groups and training backgrounds, often to a degree comparable to high-load training (Shah et al., 2026). A meta-analysis further demonstrated that BFR combined with resistance training significantly enhances lower limb maximal strength in athletes (Deng et al., 2025). Beyond these general effects, recent studies have specifically examined BFR training in sprinters. Chen et al. (2021) reported in a randomized controlled trial that running exercise combined with BFR improved both muscle strength and sprint performance in sprinters. Thakur et al. (2026), in a literature review specifically focused on 100-m sprinters, concluded that low-intensity BFR training induces hypertrophic and strength adaptations comparable to high-load training while minimizing mechanical strain and fatigue. Furthermore, a systematic review and meta-analysis confirmed that high-load BFR significantly improves muscle strength, power, and speed in athletes (Su et al., 2025). By increasing metabolic stress and accelerating type II fibre recruitment, BFR may provide a potent stimulus for improving explosive strength and anaerobic capacity even with low external loads.

Combining FST with BFR theoretically allows you to benefit from both training methods simultaneously. FST provides multi-dimensional improvement in athletic ability and optimization of movement patterns, while BFR compensates for the shortcomings of low-load training by increasing metabolic load, promoting muscle strength and hypertrophy. The study by Da Silva-Grigoletto et al. (2020) found that FST combined with BFR may maximize the synergistic adaptation of strength, hypertrophy, and functional performance through dual mechanical and metabolic stimulation. In a study of older adults, 6 weeks of FST combined with BFR significantly reduced myostatin levels (a marker of muscle atrophy) by 30.7%, increased follistatin levels by 13.7%, and showed more significant improvements in grip strength and shoulder girdle muscle strength (Pazokian et al., 2022),Another study found that FST combined with BFR could more effectively reduce the C-terminal aggregate protein fragment (CAF), a marker of neuromuscular junction degeneration, by 26.8%, and improve muscle mass indicators (Bigdeli et al., 2020). However, existing research on FST combined with BFR mainly focuses on functional rehabilitation in the elderly and rehabilitation treatment for injured patients. Research on improving lower limb specific abilities in young athletes, especially sprinters, remains scarce (Bigdeli et al., 2020; Huang and Wang, 2023; Pazokian et al., 2022). Sprinters have significantly higher requirements for lower limb strength and explosive power than the general population, and the quality of their movements directly affects running economy and athletic performance (Thibault et al., 2025; Slawinski et al., 2010). At the same time, college sprinters are in a critical period of rapid development of their athletic ability and have high potential for training adaptation (Nuell et al., 2019).

Therefore, this study aims to investigate the effects of 8 weeks of FST combined with BFR training on lower limb muscle strength, explosive power, and movement quality in male university students running the 100m and 200m sprints, providing empirical evidence for the scientific training of sprinters. We hypothesize that, compared to FST alone, FST combined with BFR can produce a more significant improvement in lower limb strength and explosive power while maintaining improved movement quality, by enhancing metabolic stimulation and fast-twitch muscle fibre recruitment.

## 2 Methods

### 2.1 Participants

The sample size was estimated based on similar experiments (Zuo et al., 2022). Furthermore, *a priori* power analysis using G*Power (version 3.1.9.2) for a two−way repeated−measures ANOVA (within−between interaction) indicated that, with a medium−to−large effect size (Cohen’s f = 0.3), a statistical power of 0.8, and a significance level of α = 0.05, at least 12 participants were required in each group. Cohen’s f is the standard effect size metric for this design in G*Power; it is related to partial η² by the formula η² = f²/(1+f²). The chosen f = 0.3 corresponds to a partial η² of approximately 0.08, which is considered a medium−to−large effect.

40 male sprinters from the Sports Science centre at Fujian normal university accepted the recruitment. The inclusion criteria are as follows: (1) Since the metabolic requirements of 400-meter sprinting are fundamentally different from those of 100-meter and 200-meter sprinting, this study only recruited 100-meter and 200-meter sprinters as subjects. The reason is that 100-meter and 200-meter sprints mainly rely on the phosphagen system (ATP-PCr system), which contributes about 79% and 72-75% of energy, respectively (Duffield et al., 2004), The 400-meter sprint, on the other hand, significantly increases the reliance on the glycolytic system, with the aerobic system contributing 41% to 59% (Duffield et al., 2005). (2) Physically healthy and without any sports injuries in the past 3 months, (3) Having systematically trained in sprinting for at least 1 year, and (4) Not having alcoholism, smoking addiction, or drug dependence.

**Figure 1:**
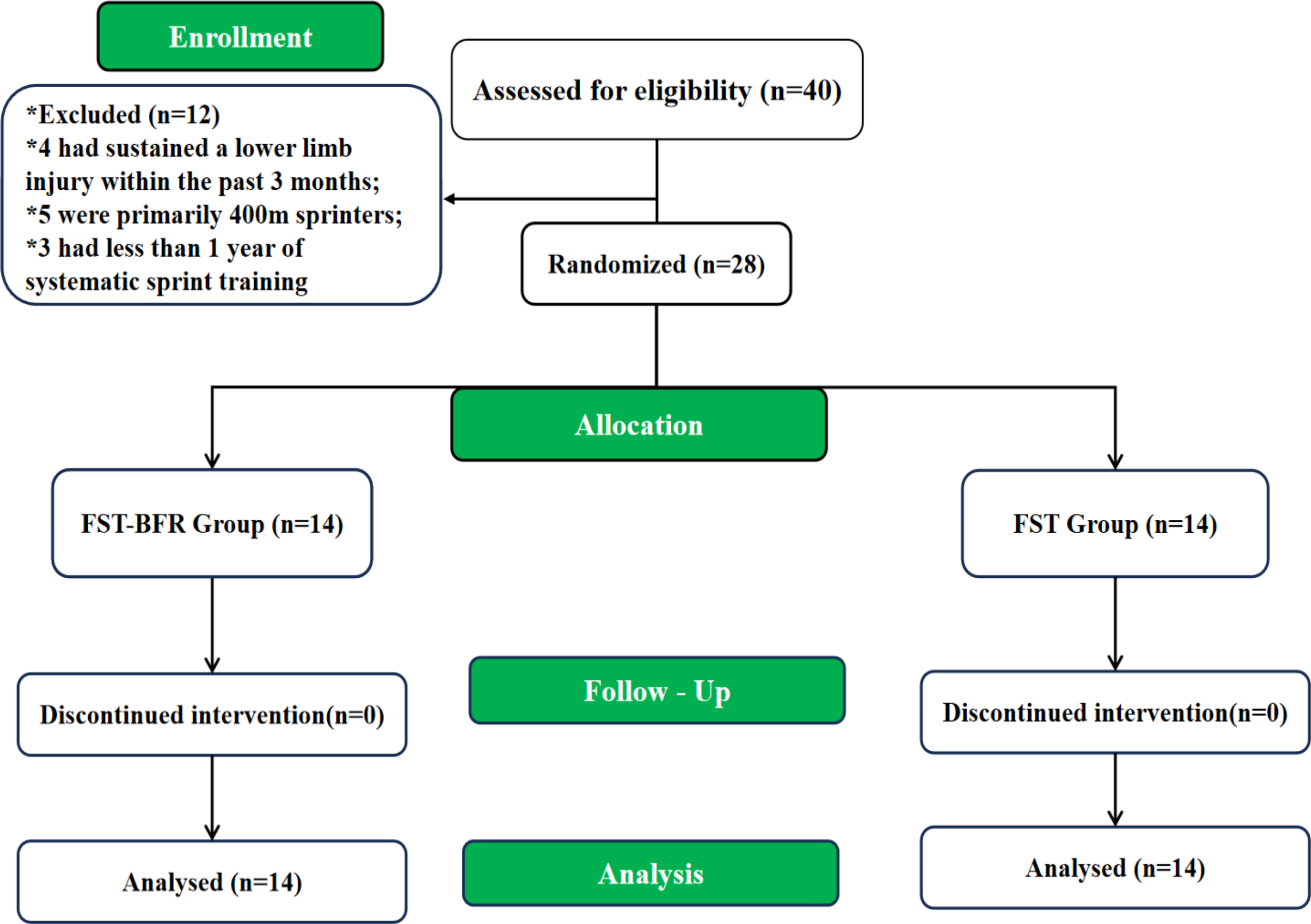
Study design flow.

A total of 40 male sprinters were initially recruited. Of these, 12 were excluded because they did not meet the inclusion criteria (e.g., 4 had sustained a lower limb injury within the past 3 months, 5 were primarily 400m sprinters, and 3 had less than 1 year of systematic sprint training). No participants dropped out during the 8week intervention (**Figure 1**). This study was approved by the Ethics Committee of Fujian Normal University (Ethics approval number: FJNU20251010). All participants were adults (aged ≥18 years) who were fully informed of the study purpose and procedures and provided voluntary written informed consent prior to enrolment, and all participants provided informed consent. Eligible participants were randomly assigned to either the FST−BFR group (n = 14) or the FST group (n = 14) using a 1:1 allocation ratio. Randomisation was conducted as follows: the allocation sequence was computer-generated by an independent statistician using www.randomization.com with a 1:1 allocation ratio. Allocation codes were placed in opaque, sequentially numbered, sealed envelopes prepared by a researcher not involved in training delivery or outcome assessment. Following baseline testing, a separate research assistant opened each envelope in the presence of the participant to assign group allocation, thereby ensuring allocation concealment. Assessor blinding was maintained by ensuring that all pre- and post-intervention testers were fully independent of the intervention team and remained unaware of participant group assignment throughout the study. Participants were instructed not to disclose their group allocation to the testers. The study flow diagram is presented in **Figure 1**.

### 2.2 Study design

An assessor-blinded experimental design was employed to reduce intervention bias. Participants were assessed for pre- and post-intervention indicators by testers independent of the experimental procedures. Furthermore, to minimize the impact of motor differences, all participants attended a familiarization session before the formal experiment to familiarize themselves with the testing and intervention procedures.

The 8-week FST training protocol consisted of 3 sessions per week and was divided into two phases. Phase 1 (weeks 1–4): Exercises were performed on unstable surfaces with the BOSU ball dome-side up. External load started with body weight only and was gradually increased by adding dumbbells when participants demonstrated stable movement patterns. The use of unstable surfaces (BOSU ball) was grounded in the theoretical rationale that unstable-surface training enhances neuromuscular co-activation and multi-planar motor control relevant to the acceleration and ground contact phases of sprinting (Behm and Anderson, 2006; Guler et al., 2021). Phase 2 (weeks 5–8): Instability was increased by inverting the BOSU ball dome-side down. External load continued to be adjusted individually to maintain training intensity while preserving movement quality under the increased instability challenge. Training load was progressed individually using the following objective criterion: when a participant could complete 3 sets × 12–15 repetitions of a given exercise with stable movement mechanics across two consecutive sessions, the supervising coach increased the external load by 0.5–2 kg (dumbbell) or upgraded the resistance band level at the next session.

Training load was quantified using the session rating of perceived exertion (sRPE). Given the multi-joint, unstable-surface nature of FST with low external loads (bodyweight, light dumbbells, resistance bands), traditional load quantification methods are not applicable. Participants rated their perceived exertion on the Borg CR-10 scale 15–30 minutes after each session, and sRPE was calculated as CR-10 score × session duration (minutes). The sRPE method has been validated for functional training (Falk Neto et al., 2020; Neris et al., 2023). Mean sRPE values over the 8-week intervention were 6.8 ± 0.7 for the FST-BFR group and 6.5 ± 0.5 for the FST group, with no significant difference between groups, confirming that internal training loads were well matched.

During inter-set rest periods (1–2 minutes), the FST-BFR group fully deflated cuff pressure to 0 mmHg, with cuffs re-inflated to the prescribed 50% AOP immediately before the commencement of each subsequent set. All participants were required to train 3 times per week for 60 minutes each session (including a 10-minute warm-up) over the 8-week period. To minimise the impact of warm-up bias, participants jogged slowly on a treadmill for 5 minutes, followed by approximately 5 minutes of dynamic stretching (Walking Knee Hug, World’s Greatest Stretch, Side Lunge Squat, Lunge Squat, and Straight Leg Raise). Both groups followed the same training regimen, and **Table 1** provides the specific functional training programme.

**Table 1:** Training program.

Cycle	Action	Repeat	Set	Intermittent
Phase 1 (Training plan for 1–4 weeks)	Squats with resistance bands on balance discs	12-15	3	1-2 min
	Balance disc single-leg squat	12-15	3	1-2 min
	Side lunge with resistance band on balance disc	12-15	3	1-2 min
	Single-leg standing hip flexion lift	12-15/per	3	1-2 min
	Swiss ball supine knee bend	12-15/per	3	1-2 min
	Suspension Lift	12-15	3	1-2 min
	Supine hip flexion lift	12-15/per	3	1-2 min
Phase 2 (5–8 weeks)	BOSU ball with resistance band squat	12-15	3	1-2 min
	BOSU ball dumbbell single-leg squat	12-15	3	1-2 min
	BOSU ball single-leg supine glute bridge	12-15/per	3	1-2 min
	Balance disc single-leg standing hip flexion lift	12-15/per	3	1-2 min
	TRX supine knee tuck	12-15	3	1-2 min
	Suspension Lift	12-15	3	1-2 min
	Swiss ball supine hip flexion lift	12-15/per	3	1-2 min

All participants were in−season sprinters preparing for collegiate-level competitions. Their coaches required them to maintain consistent sleep schedules and daily routines throughout the intervention period. In addition, dietary intake was largely standardised because the athletes followed team−provided meal plans in the university canteen during the competitive preparation phase. Therefore, major differences in sleep, daily routines, and diet between the two groups were unlikely. Nevertheless, we did not formally monitor these factors using objective tools, which we acknowledge as a limitation (see Limitations section).

### 2.3 Blood flow restriction pressure measurement

**Figure 2:**
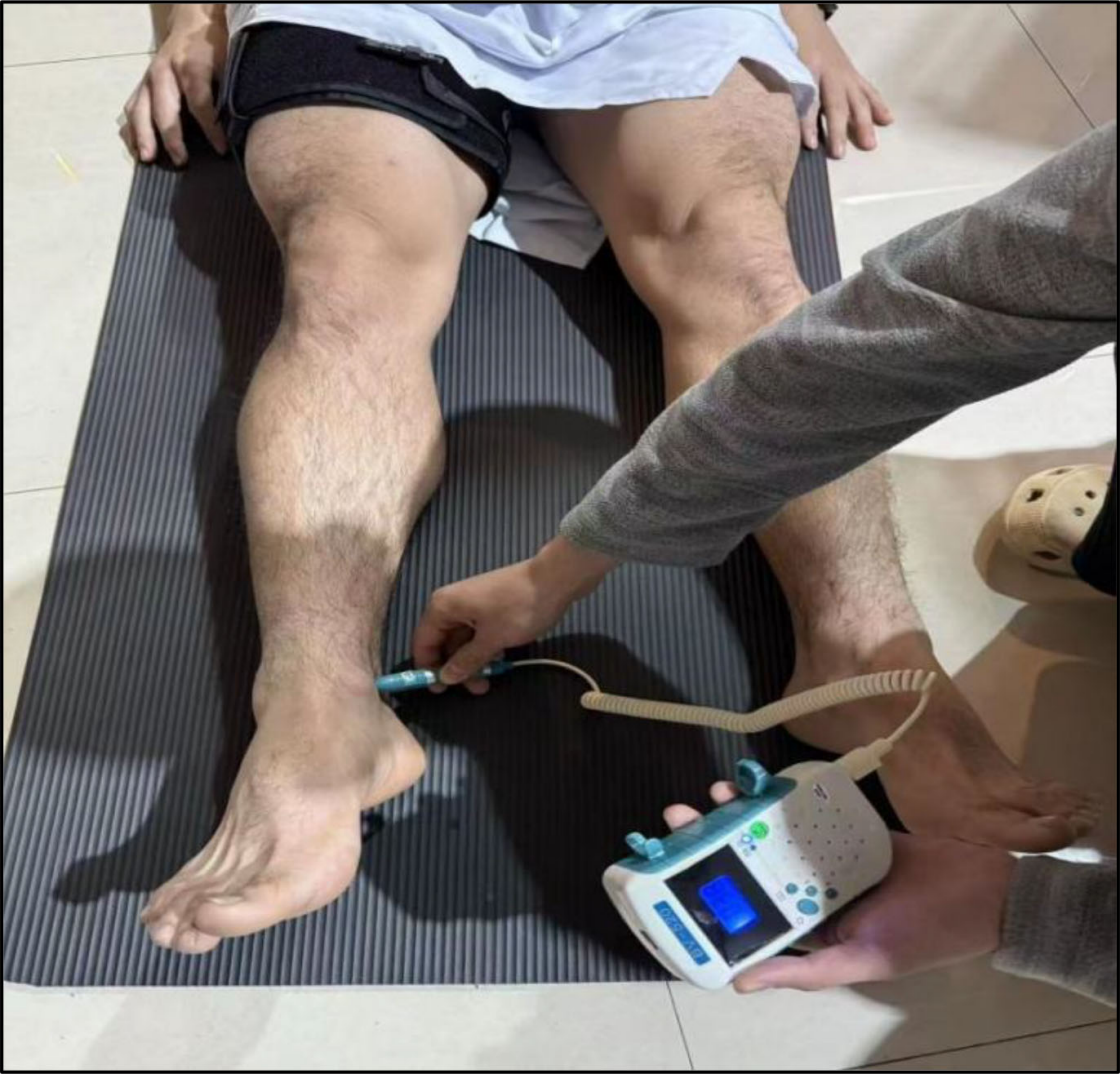
BFR pressure measurement process.

The FST-BFR group wore blood flow restriction devices bilaterally on both legs throughout the training sessions. The BFR pressure measurement procedure was as follows: after lying supine on a mat for 5–10 minutes, participants had a nylon cuff (10 cm wide, 108 cm long) placed on the most proximal portion of the thigh, positioned at a standardised distance from the inguinal crease. The mean proximal thigh circumference (measured 3 cm distal to the inguinal fold) was 55.20 ± 2.04 cm in the FST-BFR group and 52.91 ± 4.50 cm in the FST group, with no significant difference between groups (p = 0.20). The cuff-width-to-limb-circumference ratio was within the range recommended by BFR operational consensus guidelines (Loenneke et al., 2012). Ultrasound coupling gel was applied and a BV-520 handheld Doppler probe was placed over the posterior tibial artery at the posteromedial aspect of the medial malleolus (**Figure 2**). Cuff pressure was slowly increased until the Doppler signal was completely eliminated; this pressure was recorded as the arterial occlusion pressure (AOP), defined as the minimum cuff pressure required to fully eliminate the Doppler signal, recorded to the nearest 10 mmHg. Pressure was then reduced by 10 mmHg to verify signal reappearance as a safety check, but the AOP value was defined and recorded at the point of initial signal elimination. This procedure was repeated on the left thigh to determine AOP for each limb separately, ensuring symmetrical cuff placement. Considering that the safe range for cuff pressure is typically between 40% and 80% AOP, and that FST emphasises movement quality, excessively high occlusion pressure may elevate metabolic stress and compromise movement execution. Therefore, cuff pressure was individually set at 50% AOP for each leg (Patterson et al., 2019). An intermittent inflation protocol was employed: cuffs were continuously inflated during each set, fully deflated to 0 mmHg during inter-set rest periods (1–2 minutes), and re-inflated to the prescribed 50% AOP immediately before the start of each subsequent set. AOP was assessed once at baseline, and the same absolute pressure was maintained across all training sessions throughout the 8-week intervention period.

### 2.4 Indicator test

The test is conducted in two phases. The first phase includes isokinetic muscle strength and anaerobic power tests, with the remaining indicators tested at least 24 hours later. All participants are required to warm up with 5–10 minutes of stationary cycling before each test.

#### 2.4.1 Isokinetic muscle strength of the knee joint test

The strength of the dominant knee flexor and extensor muscles was assessed using a HUMAC NORM 2009 dynamometer. Equipment parameters: seat rotation 40°, tilt 0°, backrest 70–85°; force gauge angle 40°, tilt 0°, height 6, aligned with lateral femoral epicondyle; knee range of motion 0–90°. Peak torque (N·m) was measured at 60°·s^-^¹ and 300°·s^-^¹. Participants performed 4 practice repetitions at 60–80% of maximum, rested 1 min, then performed 5 maximal repetitions with verbal encouragement. The test-retest reliability of the HUMAC NORM is acceptable to good [intraclass correlation coefficient (ICC) = 0.74–0.89; Habets et al., 2018]. The standard error of measurement (SEM) for peak torque ranges from 3.7 to 11.3 N·m (de Araujo Ribeiro Alvares et al., 2015).

#### 2.4.2 Anaerobic power test

Anaerobic capacity was assessed using the Wingate Anaerobic Test (WAnT) on a mechanically braked cycle ergometer (Monark 894E, Sweden) (Zupan et al., 2009). Seat height was adjusted so that knee flexion was ≤5° at full extension. After a 3-min warm-up at 50 W (60–100 rpm), participants performed two 5-s all-out sprints against a resistance of 7.5% body weight, with 1 min active recovery (50 W) between sprints. Following 2 min of rest, participants pedalled at maximal speed; with <1 s remaining, the resistance was applied. Strong verbal encouragement was given throughout the 30-s test. Peak power (PP) was defined as the highest mean power output over any consecutive 5-second interval within the 30-second test; average power (AP) was the mean power output across the full 30-second effort; and minimum power (MinP) was the lowest mean power output over any consecutive 5-second interval. Test-retest reliability of the Wingate test is good to excellent (ICC = 0.89–0.98) with coefficients of variation (CV) of 3–6% (Jaafar et al., 2014).

#### 2.4.3 Lower limb explosive power test

Squat jump (SJ) and countermovement jump (CMJ) were measured using the OptoJump system (Optojump, Microgate, Italy) (Çetin et al., 2024). Jump height was derived from flight time at a sampling frequency of 1000 Hz. Participants performed each jump with hands on hips; three attempts were recorded and the highest value was retained for analysis. The SJ began from a stationary semi-squat position (90° knee flexion), with participants maintaining a 3-second static pause before takeoff. This pause was employed to dissipate stored elastic energy and eliminate the contribution of the stretch-shortening cycle (SSC) and stretch reflex, thereby isolating concentric-only force production capacity. The CMJ began from an upright standing position, with participants performing an immediate downward countermovement followed by a maximal vertical jump, thus utilising the SSC. Any jump in which active knee or hip flexion was observed during the aerial phase was discarded and repeated. The OptoJump system has excellent test-retest reliability (ICC = 0.982–0.989) with a coefficient of variation of 2.7% and a random error of ±2.81 cm (Glatthorn et al., 2011).

#### 2.4.4 Functional motion screening

The FMS was used to assess lower-limb functional movement quality (Cook et al., 2014a, Cook et al., 2014b). Four lower-limb-relevant items were selected from the standard seven-item FMS battery: Deep Squat, Hurdle Step, Inline Lunge, and Active Straight-Leg Raise. These items were chosen to focus the assessment on movement quality directly relevant to sprinting performance, and to minimise the influence of upper-limb fatigue and unrelated movement patterns on the composite score. Each movement was scored from 0 to 3 based on performance quality (3 = correct execution, 2 = compensation, 1 = inability to perform, 0 = pain). Two experienced researchers scored each movement independently; if the first attempt did not score 3, a second attempt was permitted, and the higher score of the two attempts was recorded as the final score for that movement pattern, consistent with the standard FMS scoring protocol (Cook et al., 2014a). The FMS has good to excellent reliability, with summary intraclass correlation coefficients (ICC) of 0.87 for intrarater reliability and 0.84 for interrater reliability (Cuchna et al., 2016).

#### 2.4.5 Lower quarter Y-balance test

Dynamic balance and lower-limb symmetry were assessed using the Y-Balance Test (González-Fernández et al., 2022). Participants stood barefoot on the platform, support leg with big toe aligned perpendicular to the reference line. The contralateral leg pushed the slider as far as possible in three directions (anterior, posteromedial, posterolateral) in random order. Three valid trials per direction per leg were recorded; a trial was repeated if balance was lost, the reaching foot touched the ground, or the support foot moved. Reach distances were normalized to leg length (ASIS to medial malleolus) and expressed as a percentage of leg length. The Y-Balance Test has good reliability in active populations, with ICC = 0.79–0.86, SEM = 2–4%, and minimal detectable change (MDC) = 5–11% (Foldager and Aslerin, 2023). Both legs were analysed separately. The dominant leg was defined as the preferred kicking leg.

### 2.5 Statistical analysis

Data analysis was performed using IBM SPSS 29.0. Normality was assessed using QQ plots and the Shapiro–Wilk test, and homogeneity of variance was assessed using Levene’s test. For variables that met the assumptions of normality and homogeneity of variance (i.e., all isokinetic strength, Wingate, CMJ, SJ, and YBT variables), a two-way repeated-measures analysis of variance (ANOVA) was conducted, with group (FST-BFR vs. FST) as the between-subject factor and time (pre- vs. post-intervention) as the within-subject factor. For the FMS variables (total score and 4 individual items), which did not follow a normal distribution, nonparametric tests were applied. The Wilcoxon signed-rank test was used for within-group comparisons and the Mann–Whitney U test for between-group comparisons.

All data are presented as mean ± standard deviation (M ± SD). For the two-way repeated-measures ANOVA, when a significant group × time interaction was detected, post-hoc comparisons were performed using Bonferroni correction to adjust for multiple comparisons. Effect sizes for the ANOVA are reported as partial eta squared (η²_p_), interpreted as small (0.01), medium (0.06), or large (0.14) according to conventional guidelines (Richardson, 2011). For nonparametric tests, effect sizes were calculated as (r = Z / \sqrt{N} ). For the Mann–Whitney U test, N = n_1_+n_2_; for the Wilcoxon signed-rank test, N = the sample size. Cohen’s guidelines for r were used: 0.1 = small, 0.3 = medium, 0.5 = large. Significance was set at p < 0.05.

## 3 Results

### 3.1 Baseline characteristics of the subjects and their attendance

The implementation of the training program was monitored by attendance at training sessions. This study designed a total of 672 training sessions to be attended, and participants completed 631 sessions within the 8-week training period, achieving an attendance rate of 93.90%.Regarding safety, no serious adverse events occurred during the intervention. A small number of participants reported transient sensations of cuff tightness or mild discomfort during training, which resolved immediately upon cuff deflation and did not require any modification to the protocol or result in participant withdrawal. Baseline characteristics are shown in **Table 2**.

**Table 2:** Baseline characteristics.

Index	FST-BFR group [95% CI]	FST group [95%CI]	P-value
Age (Y)	20.17 ± 0.65 [19.80, 20.55]	19.98 ± 0.39 [19.76, 20.21]	0.086
Height (cm)	176.86 ± 3.68 [174.74, 178.99]	177.14 ± 4.33 [174.64, 179.64]	0.980
Weight (kg)	71.49 ± 3.98 [69.19, 73.79]	71.54 ± 6.07 [68.04, 75.04]	0.923
BMI (kg/m^2^)	22.85 ± 0.88 [22.34, 23.36]	22.76 ± 1.26 [22.03, 23.49]	0.948
Training duration (Y)	1.84 ± 0.35 [1.64, 2.04]	2.09± 0.37 [1.88, 2.30]	0.678

### 3.2 Isokinetic muscle strength of the knee joint

The results (**Table 3**) are summarised as follows. A significant group × time interaction was observed only for the 300°·s^-^¹ isokinetic flexor strength (p = 0.013, partial η² = 0.216). However, simple effects analysis (post−hoc comparisons with Bonferroni correction) did not reveal a statistically significant difference between the FST−BFR and FST groups at post−intervention (p > 0.05), nor a significant difference in the magnitude of change between the two groups (p > 0.05). Both groups significantly improved from pre− to post−intervention (p < 0.001 for each). For all other isokinetic variables (60°·s^-^¹ extensor, 60°·s^-^¹ flexor, and 300°·s^-^¹ extensor), no significant group × time interaction was detected (p > 0.05), while a significant main effect of time was present (p < 0.001), indicating that both groups improved similarly.

**Table 3:** Parameter results of isokinetic muscle strength of the knee joint.

Parameters	Groups	Pre M ± SD	Post M ± SD	Time [p, F, η²_p_]	Group [p, F, η²_p_]	Time×group [p, F, η²_p_]
60°·s^-^¹ extensor (N·m)	FST-BFR	216.14 ± 20.30	239.21 ± 16.83***	P<0.001*** F=73.068 η²_p_=0.738	P=0.465 F=0.550 η²_p_=0.021	P=0.085 F=3.214 η²_p_=0.110
	FST	215.00 ± 19.43	230.07 ± 20.34***			
60°·s^-^¹ flexor (N·m)	FST-BFR	142.86 ± 17.41	155.71 ± 11.03**	P<0.001*** F=28.273 η²_p_=0.521	P=0.290 F=1.165 η²_p_=0.043	P=0.844 F=0.040 η²_p_=0.002
	FST	138.21 ± 14.78	150.14 ± 11.64**			
300°·s^-^¹ extensor (N·m)	FST-BFR	115.79 ± 9.30	125.93 ± 9.93***	P<0.001*** F=103.755 η²_p_=0.804	P=0.460 F=0.561 η²_p_=0.021	P=0.942 F=0.005 η²_p_=0.002
	FST	113.43 ± 7.06	123.43 ± 9.27***			
300°·s^-^¹ flexor (N·m)	FST-BFR	85.50 ± 10.70	98.07 ± 12.04	P<0.001*** F=80.250 η²_p_=0.755	P=0.333 F=0.973 η²_p_=0.036	**P=0.013^#^** F=7.169 η²_p_=0.216
	FST	84.50 ± 9.32	91.29 ± 11.08			

Nm, Newton-meter. Peak Torque values presented as M ± SD. **The time main effect was significant (P < 0.01); ***The time main effect was significant (P < 0.001). ^#^The interaction effect was significant (P < 0.05). Bold values indicate statistical significance.

### 3.3 Anaerobic power

The results (**Table 4**) showed significant main effects of time for PP and AP (all p < 0.05), indicating that both groups improved from pre- to post-intervention. Significant group × time interactions were observed for PP (p = 0.027, η²p = 0.174) and AP (p < 0.001, η²p = 0.756). Simple effects analysis further revealed that the improvement in AP was significantly greater in the FST-BFR group than in the FST group (p < 0.05). For PP, although the interaction was significant, *post-hoc* comparisons did not detect a significant difference between the two groups in the magnitude of change (p > 0.05). For MinP, neither the main effect of time (p = 0.273) nor the group × time interaction (p = 0.137) reached statistical significance; however, the partial η²p = 0.083 exceeded the conventional medium effect threshold (0.06), suggesting a potentially meaningful between-group difference that the current sample may have been underpowered to detect (observed power = 0.31). The FST-BFR group showed a mean increase of 32.67 W (+9.2%), whereas the FST group showed a slight decrease of 5.08 W (−1.4%).

**Table 4:** Results of anaerobic power test.

Parameters	Groups	Pre M ± SD	Post M ± SD	Time [p, F, η²_p_]	Group [p, F, η²_p_]	Time×Group [p, F, η²_p_]
PP (w)	FST-BFR	862.61 ± 105.76	983.69 ± 118.22***	P<0.001***, F=142.316, ƞ²p=0.846	P=0.431, F=0.640, ƞ²p=0.024	**P=0.027#,** F=5.492, ƞ²p=0.174
	FST	851.86± 88.58	933.18± 100.39***			
AP (w)	FST-BFR	593.52 ± 66.85	686.51 ± 57.33***#	P<0.001***, F=386.683, ƞ²p=0.937	P=0.379, F=0.802, ƞ²p=0.030	**P<0.001#,** F=80.511, ƞ²p=0.756
	FST	604.97 ± 40.02	639.69 ± 43.15			
MinP (w)	FST-BFR	357.09 ± 49.53	389.7 ± 61.80	P=0.273, F=1.256, ƞ²p=0.046	P=0.82, F=0.05, ƞ²p=0.00	P=0.137, F=2.355, ƞ²p=0.083
	FST	372.42 ± 33.48	367.34 ± 60.45			

PP, Peak Power; AP, Average Power; MinP, Minimum Power; W, watt. ***The time main effect was significant (P < 0.001). #The interaction effect was significant (P < 0.05). Bold values indicate statistical significance.

PP, Peak Power; AP, Average Power; MinP, Minimum Power; W, watt. ***The time main effect was significant (P < 0.001). #The interaction effect was significant (P < 0.05).

### 3.4 Lower limb explosive power

The results (**Table 5**) showed significant main effects of time for both CMJ and SJ (both p < 0.001), indicating that both groups improved from pre− to post−intervention. Significant group × time interactions were observed for CMJ (p = 0.005, ƞ²p = 0.267) and SJ (p = 0.010, ƞ²p = 0.228). However, simple effects analysis (post−hoc comparisons with Bonferroni correction) did not reveal a statistically significant difference between the FST−BFR and FST groups at post−intervention (p > 0.05 for both CMJ and SJ), nor a significant difference in the magnitude of change between the two groups (p > 0.05). The main group effects were not significant for either jump (p > 0.05).

**Table 5:** Results of lower limb explosive power test.

Parameters	Groups	Pre M ± SD	Post M ± SD	Time [p, F, η²_p_]	Group [p, F, η²_p_]	Time×Group [p, F, η²_p_]
CMJ	FST-BFR	55.09 ± 3.81	58.12 ± 3.13***	P<0.001***, F=136.002, ƞ²p=0.840	P=0.268, F=1.282, ƞ²p=0.047	**P=0.005^#^,** F=9.463, ƞ²p=0.267
	FST	57.19 ± 3.59	58.96 ± 3.33***			
SJ	FST-BFR	50.19 ± 3.78	53.1 ± 2.98***	P<0.001***, F=161.092, ƞ²p=0.861	P=0.281, F=1.212, ƞ²p=0.045	**P=0.010^#^,** F=7.695, ƞ²p=0.228
	FST	52.09 ± 3.32	53.96 ± 3.27***			

CMJ, Countermovement Jump; SJ, Squat Jump; cm, centimetre. ***The time main effect was significant (P < 0.001). #The interaction effect was significant (P < 0.05). Bold values indicate statistical significance.

### 3.5 FMS test

Nonparametric tests were used to analyse the FMS scores (**Table 6**). Within−group comparisons (Wilcoxon signed−rank test) showed significant improvements in the FST−BFR group for all four components and the total score (all p < 0.05). In the FST group, significant improvements were observed for Deep Squat, Inline Lunge, Active Straight−Leg Raise, and the total score (p < 0.05), but not for Hurdle Step (p = 0.157). Between−group comparisons (Mann−Whitney U test) revealed no significant differences between the two groups for any FMS variable (all p > 0.05). Effect sizes (r) for between−group comparisons were small (r = 0.00–0.15).

**Table 6:** Results of FMS.

Parameters	Groups	Pre M ± SD	Post M ± SD	Intra groups p	Inter groups p	z	Effect r
Deep Squat	FST-BFR	2.21 ± 0.43	2.57 ± 0.51	0.025*	0.458	-0.742	-0.14
	FST	2.14 ± 0.36	2.43 ± 0.51	0.046*			
Hurdle Step	FST-BFR	2.21 ± 0.58	2.50 ± 0.52	0.046*	0.453	-0.750	-0.14
	FST	2.21 ± 0.43	2.36 ± 0.50	0.157			
Inline Lung	FST-BFR	2.07 ± 0.48	2.71 ± 0.47	0.003**	1.000	0.000	0.00
	FST	2.14 ± 0.54	2.71 ± 0.47	0.005**			
Active Straight-Leg Raise	FST-BFR	1.79 ± 0.43	2.50 ± 0.52	0.002**	1.000	0.000	0.00
	FST	2.00 ± 0.56	2.50 ± 0.52	0.008**			
Total Scores	FST-BFR	8.29 ± 0.99	10.29 ± 1.07	<0.001***	0.437	-0.778	-0.15
	FST	8.50 ± 0.86	10.00 ± 0.96	<0.001***			

*The time main effect was significant (P < 0.05), **The time main effect was significant (P < 0.01), ***The time main effect was significant (P < 0.001). Bold values indicate statistical significance.

### 3.6 LQ-YBT test

The results of the Lower Quarter Y−Balance Test are presented in **Table 7**. No significant group main effects or group × time interactions were detected for any reach direction (all p > 0.05). Significant main effects of time were observed for L−Ant (p < 0.001), R−Ant (p < 0.001), L−PM (p < 0.001), R−PM (p < 0.001), L−PL (p < 0.001), and R−PL (p = 0.021).

**Table 7:** Results of LQ -YBT test.

Parameters	Groups	Pre M ± SD	Post M ± SD	Time [p, F, η²_p_]	Group [p, F, η²_p_]	Time×group [p, F, η²_p_]
L-Ant (%)	FST-BFR	60.84 ± 3.18	63.61 ± 2.81***	P<0.001***, F=85.531, ƞ²p=0.767	P=0.563, F=0.343, ƞ²p=0.013	P=0.175, F=1.943, ƞ²p=0.070
	FST	61.10 ± 4.64	64.84 ± 3.09***			
R-Ant (%)	FST-BFR	61.03 ± 5.41	64.11 ± 4.99***	P<0.001***, F=111.793, ƞ²p=0.811	P=0.417, F=0.679, ƞ²p=0.025	P=0.363, F=0.862, ƞ²p=0.032
	FST	62.75 ± 4.37	65.34 ± 4.27***			
L-PM (%)	FST-BFR	101.76 ± 7.10	110.86 ± 7.11***	P<0.001***, F=456.596, ƞ²p=0.946	P=0.511, F=0.444, ƞ²p=0.017	P=0.437, F=0.623, ƞ²p=0.023
	FST	99.88 ± 9.68	108.33 ± 10.85***			
R-PM (%)	FST-BFR	100.61 ± 2.90	111.78 ± 6.29***	P<0.001***, F=31.109, ƞ²p=0.545	P=0.440, F=0.616, ƞ²p=0.023	P=0.515, F=0.436 ƞ²p=0.017
	FST	100.43 ± 3.40	109.23 ± 10.70**			
L-PL (%)	FST-BFR	98.09 ± 5.68	107.25 ± 6.62***	P<0.001***, F=373.475, ƞ²p=0.935	P=0.94, F=0.00, ƞ²p=0.00	P=0.799, F=0.066, ƞ²p=0.003
	FST	97.96 ± 10.33	106.89 ± 11.48***			
R-PL (%)	FST-BFR	99.70 ± 5.98	108.30 ± 5.35***	P=0.021*, F=6.069, ƞ²p=0.189	P=0.131, F=2.427, ƞ²p=0.085	P=0.798, F=0.067, ƞ²p=0.03
	FST	101.46 ± 9.98	108.42 ± 11.20***			

LQ-YBT, Lower Quarter Y-Balance Test; Ant, anterior; PM, posteromedial; PL, posterolateral;L/R, lift/right. Normalized to leg length (%). *The interaction effect was significant (P < 0.05); **The time main effect was significant (P < 0.01);***The time main effect was significant (P < 0.001). Data are presented as Mean ± SD with 95% confidence intervals. #The interaction effect was significant (P < 0.05). Bold values indicate statistical significance.

## 4 Discussion

The aim of this study was to verify whether the combination of BFR and FST had additional benefits on lower limb muscle strength, explosive power, and movement quality in male college sprinters. After 8 weeks of intervention, the only outcome that showed a significantly greater improvement in the FST−BFR group compared with the FST group was AP during the Wingate test (p < 0.001, η²p = 0.756). For PP, 300°·s^-^¹ isokinetic knee flexor strength, CMJ, and SJ, although significant group × time interactions were observed, simple effects analysis did not detect a statistically significant difference between the two groups at post−intervention. No significant interaction effects were observed for the Y−Balance test, FMS, or other isokinetic variables (all p > 0.05). Thus, the addition of BFR to FST provided a clear additional benefit only for average power, while its effects on other strength and power measures were limited.

### 4.1 Lower limb muscle strength

To the best of our knowledge, no previous studies have examined the effects of FST combined with BFR on lower limb muscle strength in male college sprinters. After 8 weeks of intervention, both groups showed significant improvements over time in all isokinetic measures at 60°·s^-^¹ and 300°·s^-^¹, indicating that FST alone effectively enhances knee muscle strength. A significant group by time interaction was observed only for the 300°·s^-^¹ flexor strength, p = 0.013 and η²p = 0.216. However, simple effects analysis did not reveal a statistically significant difference between the two groups at post−intervention, nor a significant difference in the magnitude of change. Therefore, the addition of BFR did not provide a measurable additional benefit for isokinetic muscle strength beyond FST alone.

Our findings differ from previous studies in older adults and clinical populations, which reported that FST combined with BFR improved muscle strength more than FST alone (Bigdeli et al., 2020; Pazokian et al., 2022; Huang and Wang, 2023). Several factors may explain this discrepancy. The participants in our study were young, well−trained sprinters with relatively high baseline strength levels. In such a population, the potential for further strength gains is limited, and any additional effect from BFR may be too small to detect. In contrast, older adults and patients with knee osteoarthritis typically have lower baseline strength and may respond more readily to additional metabolic stress. The growing development of analytical healthcare infrastructures for knee rehabilitation further supports the potential of BFR-based protocols in such clinical populations (Bačić et al., 2026).

Another consideration is the low external load inherent to our FST protocol. The training was performed on unstable surfaces such as BOSU balls and balance discs, using body weight, light dumbbells, or resistance bands as the primary load. Although unstable surface training can enhance neuromuscular activation, it also reduces maximal force output compared with stable conditions (Behm and Anderson, 2006). This reduction in mechanical tension may have attenuated the hypertrophic stimulus, even when BFR was applied. Schoenfeld (2010) argued that mechanical tension is the most important mechanism for muscle growth, and that this growth process occurs during the transmission of mechanical force. When tension is low, additional metabolic stress from BFR may not be sufficient to produce extra strength gains. The cuff pressure was set at 50% of arterial occlusion pressure to prioritise movement quality. Higher pressures might produce greater metabolic stress (Patterson et al., 2019), but whether this would translate into additional strength gains remains unknown. We did not measure physiological markers such as muscle oxygenation or hormonal responses, and therefore the underlying mechanisms remain speculative in this study.

### 4.2 Lower limb explosive power

Explosive power depends not only on muscle volume but also on neural factors such as motor unit recruitment rate, synchronization, and firing frequency (Maffiuletti et al., 2016). FST involves multi−joint, whole−body exercises that may enhance neuromuscular activation and movement efficiency (La Scala Teixeira et al., 2019), and activate trunk and stabilizing muscles to facilitate force and energy transfer (Bao et al., 2025; Liao et al., 2019). In the present study, both groups showed significant improvements over time in CMJ and SJ, indicating that FST alone effectively enhances lower limb explosive power. Significant group by time interactions were observed for both CMJ and SJ, but simple effects analysis did not reveal a statistically significant difference between the two groups at post−intervention, nor a significant difference in the magnitude of change. Therefore, the addition of BFR did not provide a measurable additional benefit for CMJ or SJ beyond FST alone. This finding is consistent with a recent meta−analysis of 15 studies in athletes, which reported that blood flow restriction combined with resistance training does not offer additional benefits for jumping ability compared with traditional resistance training (Deng et al., 2025).

For anaerobic capacity measured by the Wingate test, the FST−BFR group showed a significantly greater improvement in AP compared with the FST group. No such between−group differences were observed for PP or MinP. The mechanism underlying this selective improvement in AP is not directly revealed by our data. However, one plausible explanation is that sustained power output over 30 seconds relies heavily on glycolytic metabolism and fatigue resistance, whereas PP and CMJ/SJ depend more on the phosphagen system and rapid neural activation (Maffiuletti et al., 2016). BFR is known to increase metabolic stress through local hypoxia and metabolite accumulation, which may preferentially enhance glycolytic capacity and type II fibre recruitment (Pearson and Hussain, 2015). These adaptations could be more beneficial for maintaining power over repeated contractions than for single maximal efforts. Nevertheless, we did not measure blood lactate, muscle oxygenation, or enzyme activity, so this interpretation remains speculative. Given that only AP showed a statistically significant between−group difference, this result should be interpreted cautiously until replicated in future studies.

### 4.3 Functional ability and lower limb dynamic balance

Intra−group differences showed significant improvements in Deep Squat, Inline Lunge, Active Straight−Leg Raise, and total FMS score in both the FST−BFR and FST groups. Improved neural mechanisms may facilitate the activation of synergistic muscles and reduce the activation of antagonistic muscles, thereby enhancing motor unit synchronicity, which contributes to improved movement quality (Enoka, 1997; Liao et al., 2019; La Scala Teixeira et al., 2019). Meanwhile, significant intra-group variations in the hurdle step were observed in the FST-BFR group. In the hurdle step test, the stability of the supporting leg plays a crucial role in completing the movement (Cook et al., 2014a), BFR may indirectly improve single-leg stability by promoting muscle strength growth. Studies have found that female patients with ankle instability showed significant improvement in muscle strength and dynamic balance around the ankle joint after BFR combined with rehabilitation training (Mahmoud et al., 2023). However, between−group comparisons revealed no significant differences in any FMS variable (all p > 0.05). Although some studies have shown that BFR helps improve muscle stability (Kong et al., 2022), the improvement in movement quality is influenced by multiple factors, including flexibility, suppleness, and stability (Liao et al., 2019). Therefore, further research may be needed to understand the extent of these influences.

A difference of more than 4 cm between the left and right sides of the YBT is considered to potentially increase the risk of injury (Xie et al., 2025). Longer YBT test distances indicate a better ability to stabilise the body in different movements (Fullam et al., 2014). In this study, significant main effects of time were observed in both groups for all reach directions (anterior, posteromedial, and posterolateral) on both legs, indicating that both training methods improved lower limb dynamic balance. Similar studies have shown that patients after anterior cruciate ligament reconstruction improved YBT scores after 12 weeks, regardless of whether they received neuromuscular electrical stimulation or BFR training (Kong et al., 2022). Li et al. (2023) reported that improved YBT results in such patients may be related to enhanced hip muscle strength induced by blood flow restriction (BFR). In contrast, our study did not observe any significant group × time interactions for YBT indices, suggesting that the addition of BFR did not confer additional benefits beyond FST alone in this population of male sprinters.

### 4.4 Limitation

Several limitations should be acknowledged. First, the sample size was small (28 participants, 14 per group). Although it was sufficient to detect a medium−to−large interaction effect (Cohen’s f = 0.3) for a single outcome, the study examined multiple outcomes and interactions simultaneously, which reduces statistical power and increases the risk of Type II errors. Therefore, non−significant findings should be interpreted with caution, and future studies with larger samples are needed.

Second, the sample consisted exclusively of male college sprinters, limiting the generalisability of the findings to female athletes, elite competitive sprinters, or recreational exercisers. Additionally, although all participants were recruited from the same university sprint programme and followed a unified team training schedule during the intervention, concurrent training load — for example, daily sprint training volume — was not objectively monitored using training logs or wearable devices. While randomisation reduces the risk of systematic between−group differences, and the high session attendance rate (93.90%) confirmed good adherence to the supervised protocol, we cannot fully exclude the possibility of meaningful variation in individual external training exposure.

Third, although sleep and dietary patterns were broadly standardised through team-provided meal plans and coach-regulated routines during the competitive preparation phase, these variables were not formally assessed using objective instruments, representing a further uncontrolled factor. Fourth, the 8-week intervention period may be insufficient to capture long-term structural and neuromuscular adaptations. Future research should extend the intervention duration, include female college sprinters, recruit participants from a single team operating under a strictly controlled training schedule, and incorporate objective monitoring tools — such as training logs or wearable devices — to document concurrent training load and lifestyle variables throughout the intervention period, thereby strengthening both the internal and external validity of findings.

The absolute external load values (e.g., dumbbell weight) used in each training session were not systematically recorded for all participants. While sRPE data confirmed comparable internal training loads between groups, the absence of external load records means we cannot confirm with precision that both groups trained under equivalent mechanical loading throughout the intervention. Future studies should systematically document session-by-session external load parameters.

Another limitation of this study is that AOP was measured only once at baseline, and a fixed absolute cuff pressure was used during the 8-week intervention without periodic reassessment. As training progresses, vascular adaptation (e.g., changes in vascular compliance) and thigh circumference can cause relative AOP (%AOP) to drift over time, thus affecting the dose-response relationship of the intervention. The BFR consensus guideline (Patterson et al., 2019) recommends reassessing AOP every 2–4 weeks, and future studies should follow this recommendation to improve the accuracy of intervention dose control.

## 5 Conclusion

8 weeks of FST, with or without BFR, was associated with significant improvements over time in 60°·s^-^¹ knee isokinetic flexor strength, FMS total score, and YBT performance in male college sprinters. However, in the absence of a no-intervention control group, these improvements cannot be directly attributed to the FST programme itself, as contributions from ongoing sprint training, maturation, or repeated-testing effects cannot be excluded. Regarding the added effect of BFR, a significant additional benefit was observed only for average power during the Wingate anaerobic test. For other measures — including 300°·s^-^¹ knee isokinetic flexor strength, CMJ, SJ, and peak power — no statistically significant between-group differences were detected at post-intervention despite some significant group × time interactions, suggesting that the current sample may have been underpowered to resolve these effects. Given the potential influence of concurrent team training and other unmonitored confounders, these findings should be interpreted with caution. Future studies incorporating larger samples, longer intervention periods, stricter control of confounding variables, and inclusion of female college sprinters are needed before recommending the routine integration of BFR into FST programmes for sprint athletes.

## Data Availability

The raw data supporting the conclusions of this article will be made available by the authors, without undue reservation.
